# Phylogenetic and Demographic Insights into Kuhl’s Pipistrelle, *Pipistrellus kuhlii,* in the Middle East

**DOI:** 10.1371/journal.pone.0057306

**Published:** 2013-02-26

**Authors:** Timothy C. Bray, Osama B. Mohammed, Abdulaziz N. Alagaili

**Affiliations:** KSU Mammals Research Chair, Department of Zoology, King Saud University, Riyadh, Saudi Arabia; Institut de Biologia Evolutiva - Universitat Pompeu Fabra, Spain

## Abstract

Kuhl’s pipistrelle is found from Europe and North Africa all of the way to Asia, yet studies have thus far concentrated on the western limit of its distribution. Here we form a multi-marker picture of the diversity of Kuhl’s pipistrelle at a mid point in the Arabian peninsula in an attempt to redress the western sampling bias and to represent a region from which no genetic data has thus far been presented for this species. The three Arabian Cytochrome *b* haplotypes showed a clear divergence of 19 substitutions from those found in either Europe or North Africa. Molecular dating suggests the Arabian population split from the remaining Kuhl’s somewhere between 0.7 and 1.7 million years before present around the time of a series of aridification events across northern Africa. Well supported lineages within Arabia are typical of that which may be seen after an expansion from multiple Pleistocene refugia, but may also reflect the loss of intermediate haplotypes during historical population fluctuations. A long-term population contraction coincides with climatic changes towards those conditions more typical of contemporary Arabia.

## Introduction

The Arabian peninsula holds a central position between Africa and Asia as recognised by studies on human movements [1], [2]. It represents a junction between Europe and Asia, incorporated into the distributions and migration routes of many species with a Eurasian distribution (e.g. [3]). Despite this central position, Arabia’s restricted connectivity with other land masses is reflected in high levels of endemism among vertebrates, including some ∼22% of ∼476 species found in the Arabian peninsula [4]. The break of the isthmus spanning the Bab al Mandab strait (∼0.5 million years ago (mya) [5]) effectively split Arabia from the Horn of Africa biodiversity hotspot [4]. At this point widespread species occupied a pan-Middle Eastern range and were still moving into the peninsula from Africa as recently as 6000 years before present (e.g. Dorcas gazelle [6]). Climatic variation is likely to have played an important role in driving adaptive evolution within Arabia with aridification increasing isolation and creating desert specialists (e.g. Arabian oryx; [7]). The most recent phase of this aridification is seen from 8500 years before present [8], but it is likely that conditions in Arabia mirrored those seen during the African shift towards more arid conditions as early as 2.8 mya [9].

There is a paucity of data on the eastern extents of many species that have been well studied in Europe, this is particularly true for volant organisms characterised by wide distributions (e.g. bats see [10]). As such, the contributions of Arabian populations to the diversity of these species is largely unknown but potentially interesting. For example, recent attention directed towards the Chiroptera has extended ranges into the Arabian peninsula [11] as well as prompting the description of new species across several families [12], [13]. Through their role as bioindicators of ecosystem health [14], insectivorous bat assemblages can also shed light on understudied human impacts across ecosystems such as from overgrazing and pollution.

Here we focus on Kuhl’s pipistrelle, *Pipistrellus kuhlii,* a species that is widespread across Europe, Africa and Asia [15]. Radiations in *Pipistrellus* in the Mediterranean have been attributed to allopatry as a consequence of habitat fragmentation during the Messinian salinity crisis (7.3–5.2 million years ago [16]). Kuhl’s pipistrelle has been shown to comprise two distinct haplogroups in Europe and north Africa [17], but nothing is known of its genetic composition towards the eastern Mediterranean. Currently two sub-species (*P. k. kuhlii/P. k. ikhwanius*) are described in Arabia, with very little known about the genetic structure of the population of Kuhl’s pipistrelle within the Arabian peninsula [18]. Long-distance flight and migration may result in population admixture in some bat species, but this does not exclude the existence of unique Arabian lineages, as has been seen in other species (e.g. *Rhinopoma microphyllum, R. muscatellum* [*12*]).

Taking a multi-marker approach (microsatellite, mitochondrial, and nuclear sequence) we question the importance of this overlooked region of the range of a widespread bat species *P. kuhlii* from a central position in the Arabian peninsula. Our aim is three-fold: 1) To consider the genetic diversity of the Arabian *P. kuhlii* in a phylogeographic context, 2) To identify any patterns of contemporary spatial variation or genetic structure, and 3) To assess the current and historic demography of Kuhl’s pipistrelle in Arabia. These results will extend the wider phylogeography of the Kuhl’s pipistrelle and allow the first conservation appraisal of one of Arabia’s common species.

## Methods

### Ethics Statement

Handling and euthanasia were conducted humanely according to the Institutional Animal Care and Use Committee at the University of Arkansas according to protocol number 07002 (permission granted on the 15^th^ of August 2006). Permission for sample collection was granted by the Saudi Wildlife Authority.

### Sampling

Pipistrelle bats were caught from 13 roosting sites (n = 51, 1–6 per location) between 1 and 88 km apart in the Qasim region of the Kingdom of Saudi Arabia. Bats were captured using harp traps and mist nets. Euthanasia was performed by briefly sedating the animal using xyalzine hydrochloride (Rompun®, 20 mg/ml, Bayer, Leverkusen, Germany) and then followed by intraperitoneal injection of Euthatal (Pentobarbital Sodium 200 mg/ml, Merial, Essex, UK). Animal death was inferred from absence of respiratory movement and heart beat, and when there was loss of colour in mucous membranes.

### Molecular Data

DNA extraction was performed from pectoral muscle tissue using Qiagen extraction reagents and separate spin columns (Epoch Life Science). Six microsatellite markers designed in other pipistrelle species were applied; 1–26, L-45, WW6 [19] and PIP01, PIP02, PIP05 [20]. All markers were amplified in multiplex PCR (40 cycles at 60°C annealing temperature) with the exception of 1–26 which was amplified on its own. To confirm genotypes and assess scoring error 10% of genotypes were repeated. The mitochondrial cytochrome *b* (*cytb*) gene was amplified using the Molcit/MVZ16 primers [20]. The nuclear recombination activating gene 2 (RAG2) was amplified using F1INT/R1 primers [21]. For both genes PCR was run for 40 cycles at 48°C annealing temperature. Reverse sequences were generated for confirmation.

### Phylogenetic Analysis

Sequence data was cleaned and aligned in BIOEDIT [22]. Nucleotide substitutions and translation into amino acids was performed through MEGA5 [23]. The most appropriate nucleotide substitution model to apply for the subsequent analyses was determined according to the Akaike Information Criterion (AIC [24]) using JMODELTEST [25]. Neighbour joining trees were constructed and compared between gene regions separately (1000 bootstraps) using MEGA5 [23]. Maximum parsimony haplotype networks were calculated using the TCS [26] application.

Time since divergence of the different geographically separated Kuhl’s pipistrelle lineages was calculated using sequence data through the BEAST application [27]. Both species models (Yule; Birth-death) were tested in addition to all options for the clock models (Strict, Relaxed logarithmic, Relaxed exponential, and Random local), with likelihoods compared in TRACERv1.5 (available in BEAST) over 1×10^8^ iterations. The model with the highest likelihood was run for 1×10^9^ iterations (sampling every 1×10^5^). The molecular clock was calibrated using two fossil-dated splits between two pairs of *Myotis* species; *nattereri/schaubi* whose split is dated at 6 mya, and *bechstenii/daubentonii* dated at 5 mya [28]. These species pairs were chosen due to the poor fossil record within *Pipistrellus* [29]. Sequence data for other *Pipistrellus* and *Myotis* lineages was also included in the analyses (Table S1; [30]–[33]).

### Contemporary Spatial Variation

Microsatellite loci were checked for linkage disequilibrium, null alleles, and conformation to Hardy Weinberg. Summary statistics were calculated across all loci. Cryptic population structure was investigated through the clustering algorithm STRUCTURE [34] which was used to group individuals into K homogeneous clusters (populations). We applied the Evanno *et al*. [35] approach of selection of the K-value corresponding to the mode of the ΔK distribution. We set K to vary between 1 and 5, and for each K-value we performed 20 simulations with different starting points, having a 10^5^ burn-in period followed by 10^5^ steps. For each K-value, we calculated the average and standard deviation of the log estimated likelihood [L(K)] across the 20 runs. The program was run under the admixture model, considering correlated allele frequencies. Isolation by distance between groups of individuals at roosting locations was investigated using GENEPOPv4 [36].

### Demographic History

A Bayesian approach for detecting and quantifying demographic expansion or contraction was applied in MSVAR 1.3 [37].Contemporary (N_1_) and ancestral (N_0_) population sizes and the time since the start of the demographic event are quantified under an exponential model of population size change. Run priors were log-normal and set to vary in all combinations of 4 and 5 for the parameters N_1_ and N_0_ with standard deviations of 2. Each of the eight runs was run for 1×10^7^ iterations with a thinning interval of 1×10^4^. The first 10% of each run was removed to avoid any bias of initial conditions before being analysed using in R [38]. Initially each chain was checked by eye for convergence before comparison between chains using the gelman statistic (<1.2). Median estimates were calculated from a concatenated dataset containing the outputs of all runs. The 95% confidence in posterior distributions were calculated between the 2.5% and 97.5% intervals. Generation time for the analysis was calculated using T = (*a*+*b)*/2 [39], *a* being age at maturation, *b* being longevity. Maturation and longevity were estimated at 1 and 8 years (based on; [40], [41]). A second approach, this time for the detection of recent population size changes (either bottleneck or growth; [42]) was also applied to the microsatellite data. This analysis compared 10^4^ simulated H_E_ values against the observed dataset using the Wilcoxon’s signed rank test. Under the recommendation of Peery *et al*. [43] that multistep mutations are generally underestimated, we applied only the Two Phase mutational model under the following proportions of multisteps; 0.25, 0.35, and 0.45.

## Results

### Data Generation and Sequence Characteristics

A total of 780 bp of *cytb* and 562 bp RAG2 sequence data were generated for 30 individuals. Three haplotypes were determined from *cytb* representing 10 of the 13 roost locations. Nucleotide diversity in *cytb* was 0.04, with 10 parsimony informative sites. A single haplotype was identified from 25 individuals in the RAG2 gene, with a single variable site. Sequence data was combined with both *cytb* and RAG2 sequences already available (GENBANK accession numbers for all sequences available in Table S1).

### Phylogenetic Analysis

**Figure 1:**
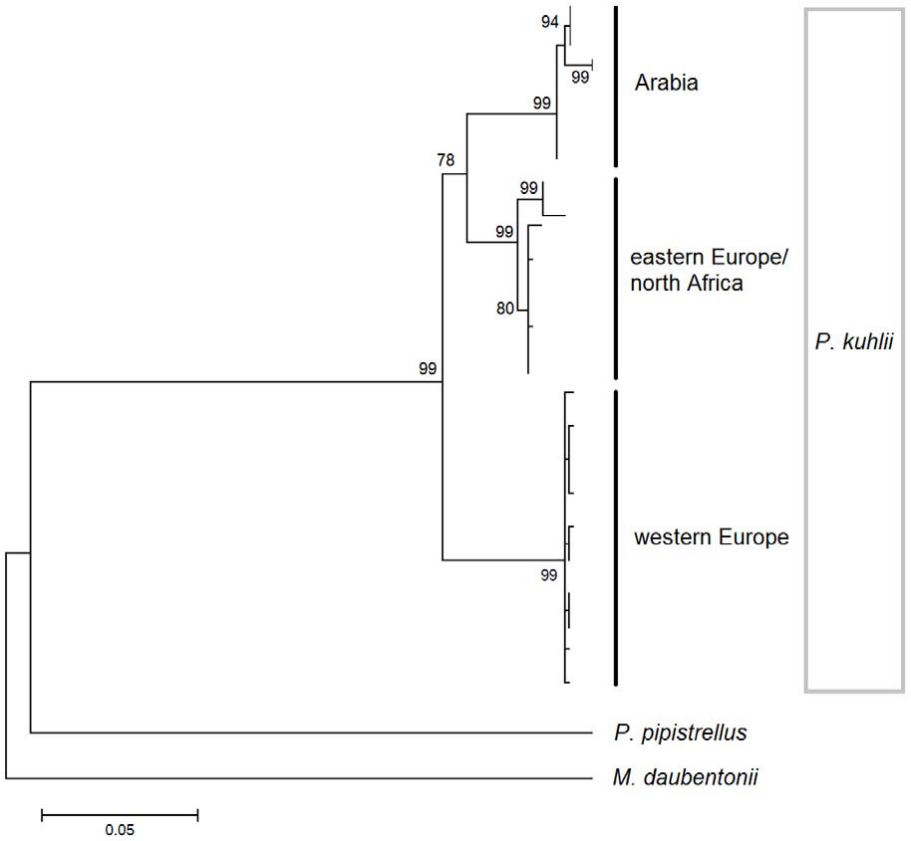
A neighbour-joining tree for the Cytochrome *b* gene for *Pipistrellus kuhlii* for the Arabian peninsula, northern Africa, and Europe [**44**]. Consensus bootstrap values are given for nodes over 75.

**Figure 2:**
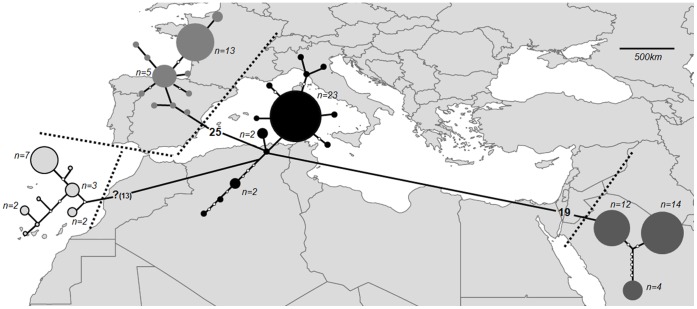
A maximum parsimony haplotype network for *Pipistrellus kuhlii* based on 780 bp* of the Cytochrome ***b*** **mitochondrial gene.** *For completeness the Canary island haplogroup is included, the information for this group is based on 515 bp and for this reason substitution distances to the other haplogroups are purely putative.

**Figure 3:**
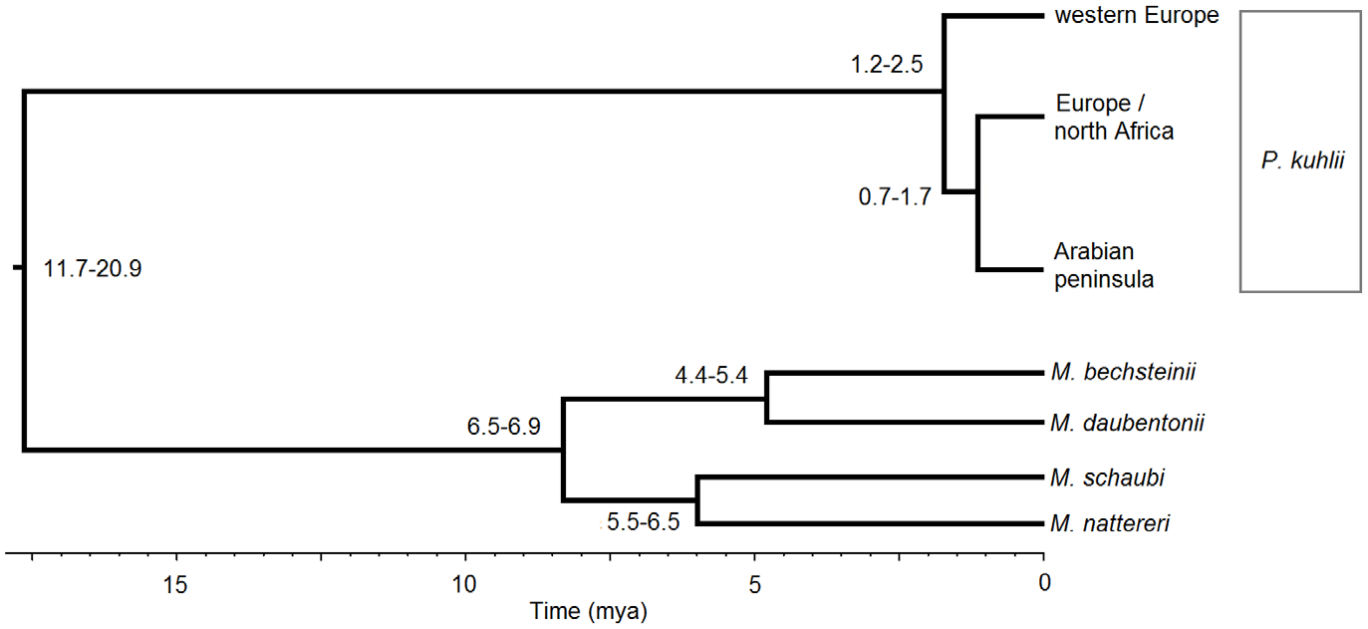
Molecular dating for the divergences within P. kuhlii using the fossil calibrated splits between *M. nattereri/schaubi* and *M. bechsteinii/daubentonii.*

For *cytb* the most appropriate mutational model was the General Time Reversible with invariable sites. A neighbour joining tree shows strong support (>99% bootstrap support) for three clusters within the Saudi Arabian samples (Figure 1), while the single RAG2 haplotype matched that previously found in central-east Morocco (data not shown, [44]). The maximum parsimony haplotype network shows that the Arabian *cytb* haplogroup is 19 substitutions removed from the closer north African/European haplogroup (Figure 2). For the Drummond and Rambaut [27] approach the highest likelihood was obtained from the Yule species model, using a strict molecular clock (likelihood = −2716). The divergence of the western European lineage is the most ancient at 1.8 mya (95% = 1.2–2.5), the split of the Arabian peninsula from north Africa/Europe being 1.2 mya (95% = 0.7–1.7; Figure 3).

### Contemporary Spatial Variation

Five of six microsatellite markers were polymorphic across 51 individuals representing 13 roost locations. No significant signal of linkage disequilibrium was detected and estimates of null allele frequencies were on or below the recommended limit (0.2; [45]). Deviations from Hardy Weinberg and summary statistics are shown in Table S2. The proportion of erroneous allele scorings was estimated at 0.04. There was no evidence of population structure (most likely K = 1) or isolation by distance (p = 0.43, Mantel test 1000 permutations) between roosting locations (data not shown).

### Demographic History

**Figure 4:**
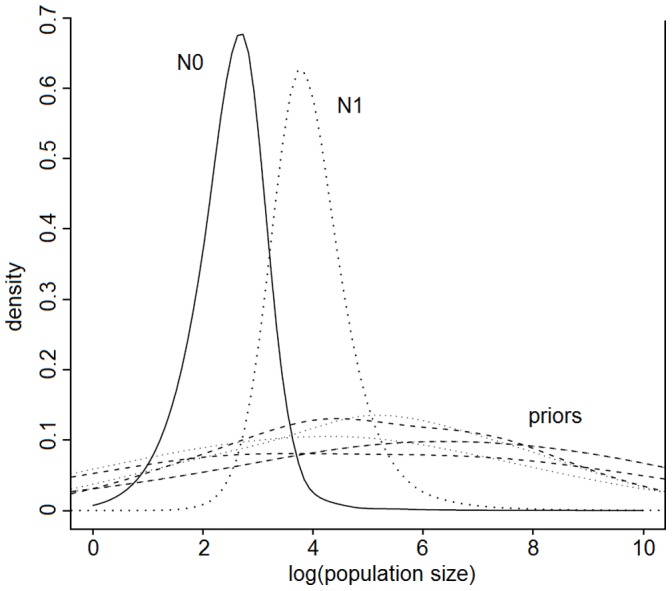
Ancestral (N_1_) and current (N_0_) population size estimates according to the Storz and Beaumont [**37**] approach. Priors are shown as dashed (N_1_) and dotted (N_0_) lines.

The Storz and Beaumont [37] method was used to identify any signal of historical population expansion or decline and to give an estimate of the magnitude and timing of this event. This approach identified a population decline (Figure 4). Current effective population size was estimated to have a median of 360 (CI; 18–2900), ancestral population size 7600 (CI: 790–1.9×10^5^). The time since the decline began was estimated at 3400 years but does not exclude the possibility of a recent dramatic decline in population size (Figure S1; CI: 56–8.1×10^5^). For comparative purposes, an approach for specifically investigating recent population expansion or decline was also applied [42]. No significant signal of either a population bottleneck or expansion was detected for any of the three mutation models (data not shown).

## Discussion

### Arabian Kuhl’s Pipistrelle in a Phylogeographic Context

It is over 2000 km between the Arabian peninsula and the closest previous sampling in this species in either Morocco or Macedonia [44]. Despite this, the Arabian haplotypes show less divergence relative to the northern African/eastern European Kuhl’s pipistrelle than is seen within Europe, with a more recent divergence between the more eastern European and the Arabian lineages compared to western Europe (Figure 3; 0.7–1.7 vs 1.2–2.5 mya). The cytochrome *b* sequence data was partitioned into three haplotypes across ten variable nucleotide positions, with one Arabian haplotype being eight substitutions removed from the remainder. This large inter-haplotype difference (average 6.7 steps) may indicate a historical division that is not evident in either the more slowly evolving nuclear RAG2, or the more rapidly evolving microsatellites. This haplotype pattern is suggestive of a period of isolation into separate refugia, or the loss of haplotypes during large fluctuations in population size. Very little information is available for Arabian refugia in other volant organisms (e.g. suggested Arabian refuge for *Calidris alpina*; [46]) and more sampling over a wider geographic extent would be needed to investigate this further.

We might expect Kuhl’s pipistrelle to follow a similar large-scale pattern of movement to that of *P. pipistrellus*, which currently occupies a similar circum-Mediterranean range [15]. The estimate for the split in Kuhl’s pipistrelle between western Europe and Arabia coincides with the time at which *P. pipistrellus* was colonising western Europe, indicating favourable conditions for northern expansion (1.6–0.9 mya [29]). But more importantly, the aridification of northern Africa is likely to have begun to curtail a major route of gene flow from the Plio-Pleistocene boundary, with an increased arid shift from around 2.8 mya [9]. However, this is not enough to explain the entire picture, as circum-Mediterranean movement would have allowed gene-flow through Europe. It is therefore likely that this north African aridification also resulted in subsequent range contraction in Kuhl’s pipistrelle throughout the region and across into Arabia.

### Contemporary Diversity

Kuhl’s pipistrelle was thought to be the only *Pipistrellus* species present in the central Arabian sampling area [18]. The genotype data confirmed that all sampled individuals comprised a single breeding population with no indication of structuring by roost or distance. This is not unexpected, as a typical foraging limit in *P. pipistrellus* in continuous good quality habitat was shown to be ∼5 km [47]. This might be expected to be greater in a patchy arid landscape which would also promote the mixing of individuals from different roosts in localised foraging areas (e.g. vegetated patches, water sources). Other bat species are recognised as commuting to feeding areas up to 25 km away [48]. Longer distance feeding movement and dispersal from natal roosts sites would also erode signal of localised genetic structure, and there is currently no information available on whether this occurs.

### Demographic History within Arabia

Population sizes are notoriously difficult to estimate in bats which often still rely on counts during roost emergence [49]. A genetic approach such as this one, allows the calculation of both contemporary and historical population size estimates. Although of restricted value independently, the combination of estimates allow consideration of population trajectories. The Storz and Beaumont [37] approach here detected the signature of a historical population decline in Arabian Kuhl’s pipistrelles (Figure 4).The median estimate for this decline dates it as ∼3.4 kya subsequent to a series of precipitation minima in the Arabian peninsula, the most recent being 4.2 kya [50]. This estimated date of decline falls during a period of suggested decline in modern human populations in southern Arabia [50] and before the onset of extremely arid conditions in north Africa associated with expansion of the Sahara desert 1500 years before present [51]. The strongly cyclical occurrence of arid episodes across Africa since the last interglacial period might suggest concurrent conditions in Arabia (latest being 9 kya [52]), perhaps contributing to the uncertainty seen in our population size estimate. Median estimates place the rate of decline at a gradual twenty-fold decrease in numbers over this time. The contemporary boundary for the 95% confidence interval of this estimate means that we cannot exclude a more recent (and therefore more rapid) population decline. The lack of evidence of a recent population bottleneck (through the Cornuet and Luikart [42] method) does, however, lend support to this not being the case. The effective contemporary population size for the study area is estimated to be up to ∼3000 individuals (i.e. numbers of breeding females). Population size estimates appear to be problematic in bats, with none available in the Kuhl’s (or any) pipistrelle. Data on roost size in *Pipistrellus* ranges from a few individuals in a small tree roost, up to around one thousand in larger caves [53], suggesting that numbers of roosts may not be a good predictor of local population size either. This being the case, our genetic estimate is likely to be of value as a reference for this bat population.

From the molecular estimates it is not possible to exclude more recent causes of bat decline in this region. The vulnerability of bats to human disturbance is well understood [14], and such problems as the deliberate poisoning of water sources remain a contemporary problem for all wildlife in Saudi Arabia (e.g. Figure S2). Perhaps most important to bats is their dependency on day roost sites [54]. Insectivorous bat assemblages have suffered from human roost disturbance and poisoning in caves elsewhere in the Middle East, such as west of the river Jordan [55]. The destruction of, and eviction from, disused traditional buildings is a more immediate threat in the Arabian sampling area (Alagaili pers. Obs.). Their utility as indicators of ecosystem degradation [14] suggests that bats can be important as bioindicators. This alone recommends the greater understanding of their habits and population dynamics in this understudied region.

### Conclusions

The molecular data presented here suggest that widespread species such as *Pipistrellus kuhlii* have extensive genetic diversity. The extent of this diversity will clearly only be apparent upon the characterisation of the entirety of their distribution. The new haplogroup described here provides the eastern-most representation of this species to date. The Arabian Kuhl’s pipistrelle appears to have been isolated from the more northern and western populations at least 0.7 mya and may have been contracting since this isolation. Although this decline is gradual we would recommend the long-term monitoring of this and other bat populations in Arabia.

The benefit of applying suites of molecular markers for estimating population size and trajectory is clear. It is also apparent that meaningful demographic signal can be retrieved even with small numbers of microsatellites as applied here, but we suspect that more accurate findings could be generated with more markers. We propose that a genetic approach such as this one will provide a useful comparison for temporal analysis in any given location for monitoring purposes. We do, however, urge caution about the limited transferability of such data between locations or species.

## Supporting Information

Figure S1 **Time since the beginning of the decline (T) including prior distributions (dashed), calculated according to the Storz and Beaumont** [**37**] **method.**
(TIF)

Figure S2 **Poison regimen applied to the stream in Wadi Lajab, Asir province, Saudi Arabia (2012).**
(TIF)

Table S1 Accession numbers for all cytochrome *b* sequences used in this study including haplotype identifiers and study of origin.
(DOCX)

Table S2 Microsatellite summary statistics for the five polymorphic loci: Expected heterozygosity (H_E_), Observed heterozygosity (H_O_), number of alleles (A), estimate of frequency of null alleles (Null), and F_IS_ (Probability of significant deviation from Hardy-Weinberg equilibrium; P<0.05 = *, P<0.01 = **, P<0.001 = ***).
(DOCX)
