# Supplementary figures and images for: Phylogenetic and Demographic Insights into Kuhl’s Pipistrelle, Pipistrellus kuhlii, in the Middle East
Source: PLoS One. 2013 Feb 26;8(2):e57306. doi: 10.1371/journal.pone.0057306 (PMC3582509; doi:10.1371/journal.pone.0057306)

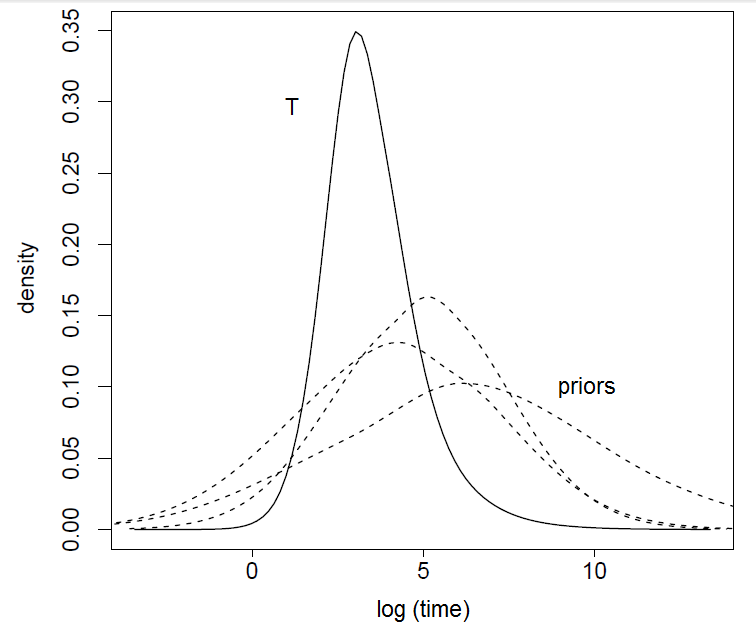

Supplement: Figure S1 — Time since the beginning of the decline (T) including prior distributions (dashed), calculated according to the Storz and Beaumont [37] method. (TIF) [file pone.0057306.s001.tif]

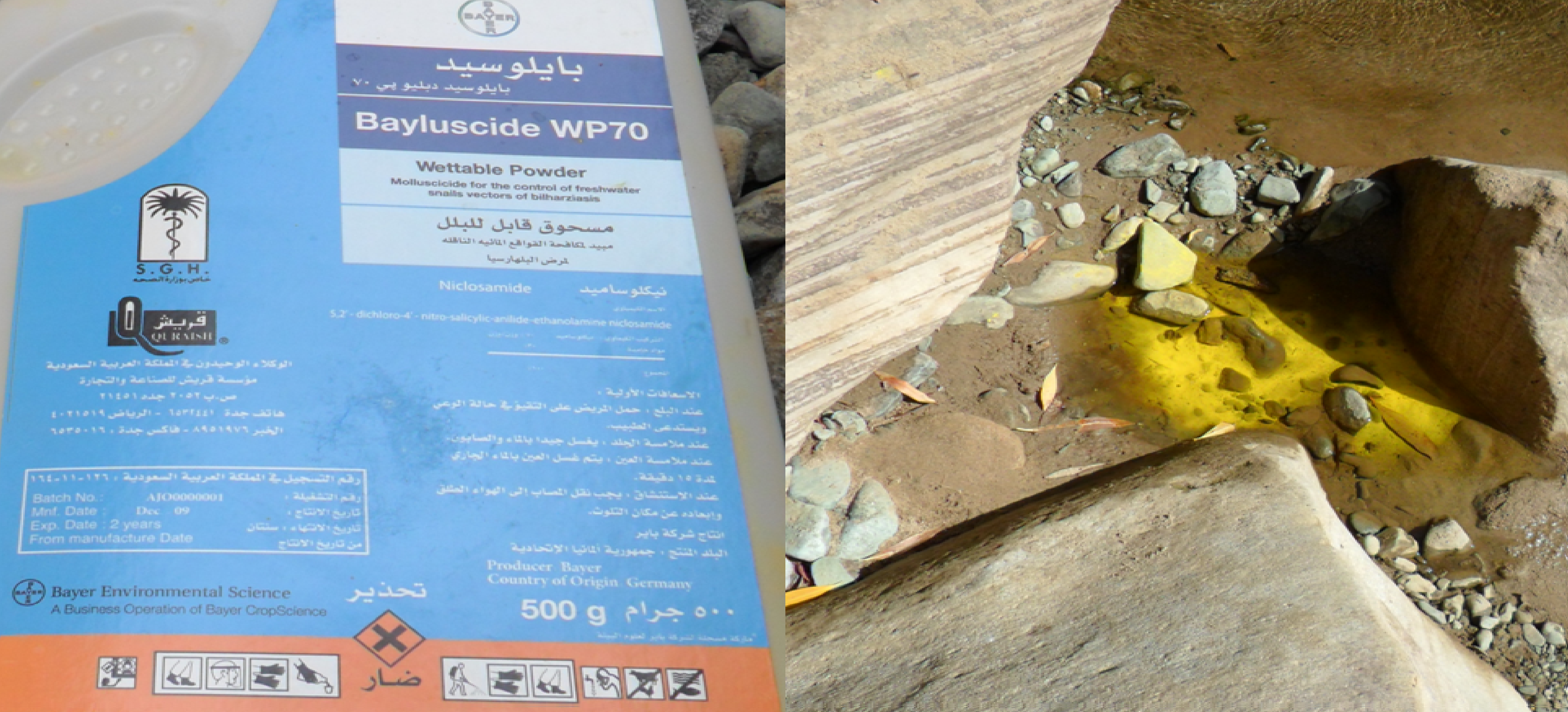

Supplement: Figure S2 — Poison regimen applied to the stream in Wadi Lajab, Asir province, Saudi Arabia (2012). (TIF) [file pone.0057306.s002.tif]
