# Supplementary material for: Phylogenetic and Demographic Insights into Kuhl’s Pipistrelle, Pipistrellus kuhlii, in the Middle East
Source: PLoS One. 2013 Feb 26;8(2):e57306. doi: 10.1371/journal.pone.0057306 (PMC3582509; doi:10.1371/journal.pone.0057306)
Supplement: Table S1 — Accession numbers for all cytochrome b sequences used in this study including haplotype identifiers and study of origin. (DOCX) [file pone.0057306.s003.docx]

| **Species** | **N** | **Origin** | **GenBank** | **Haplotype identifier** | **Reference** |
| --- | --- | --- | --- | --- | --- |
| *P. kuhlii* | 4 | Arabia | KC146388 | Ahap1 | This study |
| *P. kuhlii* | 14 | Arabia | KC146389 | Ahap2 | This study |
| *P. kuhlii* | 12 | Arabia | KC146390 | Ahap3 | This study |
| *P. kuhlii* | 2 | Canary | AJ426607 | PK1 | [30] |
| *P. kuhlii* | 1 | Canary | AJ426608 | PK2 | [30] |
| *P. kuhlii* | 7 | Canary | AJ426609 | PK3 | [30] |
| *P. kuhlii* | 2 | Corsica | HQ687493 | - | [31] |
| *P. kuhlii* | 1 | Corsica | HQ687494 | - | [31] |
| *P. kuhlii* | 6 | Corsica | HQ687495 | - | [31] |
| *P. kuhlii* | 1 | France | HQ687493 | - | [31] |
| *P. kuhlii* | 7 | France | HQ687494 | - | [31] |
| *P. kuhlii* | 1 | Greece | AJ504444 | C9 | [32] |
| *P. kuhlii* | 1 | Italy | HQ687496 | - | [31] |
| *P. kuhlii* | 1 | Italy | HQ687494 | - | [31] |
| *P. kuhlii* | 1 | Italy | HQ687495 | - | [31] |
| *P. kuhlii* | 6 | Italy | HQ687497 | - | [31] |
| *P. kuhlii* | 1 | Italy | EU360659 | C19 | [44] |
| *P. kuhlii* | 1 | Italy | EU360660 | C20 | [44] |
| *P. kuhlii* | 14 | Morocco | AJ504444 | C4 | [44] |
| *P. kuhlii* | 2 | Morocco | EU360650 | C1 | [44] |
| *P. kuhlii* | 1 | Morocco | EU360651 | C2 | [44] |
| *P. kuhlii* | 1 | Morocco | EU360652 | C3 | [44] |
| *P. kuhlii* | 3 | Spain | AJ426619 | - | [30] |
| *P. kuhlii* | 1 | Spain | DQ120841 | C14 | [44] |
| *P. kuhlii* | 1 | Spain | DQ120842 | C15 | [44] |
| *P. kuhlii* | 1 | Spain | DQ120843 | C16 | [44] |
| *P. kuhlii* | 5 | Spain | DQ120844 | C11 | [44] |
| *P. kuhlii* | 1 | Spain | DQ120845 | C12 | [44] |
| *P. kuhlii* | 1 | Spain | DQ120846 | C13 | [44] |
| *P. kuhlii* | 1 | Spain | EU360653 | C5 | [44] |
| *P. kuhlii* | 1 | Spain | EU360654 | C6 | [44] |
| *P. kuhlii* | 1 | Spain | EU360655 | C7 | [44] |
| *P. kuhlii* | 1 | Spain | EU360656 | C8 | [44] |
| *P. kuhlii* | 1 | Spain | EU360657 | C9 | [44] |
| *P. kuhlii* | 1 | Spain | EU360658 | C10 | [44] |
| *P. kuhlii* | 1 | Switzerland | DQ120847 | C17 | [44] |
| *P. kuhlii* | 1 | Switzerland | DQ120848 | C18 | [44] |
| *P. maderensis* | 2 | Canary | AJ426610 | PM1 | [30] |
| *P. maderensis* | 1 | Canary | AJ426611 | PM2 | [30] |
| *P. maderensis* | 3 | Canary | AJ426612 | PM3 | [30] |
| *P. maderensis* | 1 | Canary | AJ426613 | PM4 | [30] |
| *P. maderensis* | 1 | Canary | AJ426614 | PM5 | [30] |
| *P. maderensis* | 6 | Canary | AJ426615 | PM6 | [30] |
| *P. maderensis* | 1 | Canary | AJ426616 | PM7 | [30] |
| *P. maderensis* | 3 | Canary | AJ426617 | PM8 | [30] |
| *P. maderensis* | 1 | Canary | AJ426618 | PM9 | [30] |
| *P. maderensis* | 1 | Canary | AJ426632 | PM11 | [30] |
| *P. pipistrellus* | / | / | DQ120854 | / | [20] |
| *M. nattereri* | / | / | DQ120884 | / | [20] |
| *M. schaubi* | / | / | AF376868 | / | [33] |
| *M. bechsteinii* | / | / | DQ120899 | / | [20] |
| *M. daubentonii* | / | / | DQ120898 | / | [20] |
