# Supplementary material for: Phylogenetic and Demographic Insights into Kuhl’s Pipistrelle, Pipistrellus kuhlii, in the Middle East
Source: PLoS One. 2013 Feb 26;8(2):e57306. doi: 10.1371/journal.pone.0057306 (PMC3582509; doi:10.1371/journal.pone.0057306)
Supplement: Table S2 — Microsatellite summary statistics for the five polymorphic loci: Expected heterozygosity (HE), Observed heterozygosity (HO), number of alleles (A), estimate of frequency of null alleles (Null), and FIS (Probability of significant deviation from Hardy-Weinberg equilibrium; P<0.05 = *, P<0.01 = **, P<0.001 = ***). (DOCX) [file pone.0057306.s004.docx]

|  | H_E_ | H_O_ | A | Null | F_IS_ |
| --- | --- | --- | --- | --- | --- |
| 1-26 | 0.83 | 0.72 | 9 | 0.15 | 0.14 |
| L45 | 0.68 | 0.54 | 6 | 0.14 | 0.21* |
| pip01 | 0.62 | 0.60 | 5 | 0.13 | 0.05 |
| pip05 | 0.47 | 0.30 | 4 | 0.20 | 0.38** |
| WW6 | 0.17 | 0.19 | 2 | 0 | 0.10 |
| Average | 0.47 | 0.39 | 4.5 | - | - |
